# Ideal observer estimation for binary tasks with stochastic object models

**DOI:** 10.1088/1361-6560/ae3c53

**Published:** 2026-02-20

**Authors:** Jingyan Xu, Frederic Noo

**Affiliations:** 1 Department of Radiology, Johns Hopkins University, Baltimore, MD, United States of America; 2 Department of Radiology and Imaging Science, University of Utah, Salt Lake City, UT, United States of America

**Keywords:** area under the receiver operating characteristic (ROC) curve (AUC), Bayesian inference, dual energy CT, ideal observers, spectral optimization, stochastic object models (SOMs)

## Abstract

*Objective.* We propose a new formulation for ideal observers (IOs) that incorporate stochastic object models (SOMs) for data acquisition optimization. *Approach*. A data acquisition system is considered as a (possibly nonlinear) discrete-to-discrete mapping from a finite-dimensional object space, $x \in R^{n_d}$, to a finite-dimensional measurement space, $y \in R^{m}$. For binary tasks, the two underlying SOMs, $H_0$ and *H*_1_, are specified by two probability density functions (PDFs) $p_{0}(x)$, $p_{1}(x)$. This leads to the notion of intrinsic likelihood ratio (LR) $\Lambda_{I} (x) = p_{1}(x)/p_{0}(x)$ and intrinsic class separability (ICS), the latter quantifies the population separability that is independent of data acquisition. With respect to ICS, the IO employs the ‘extrinsic’ LR $\Lambda(y) = {\mathrm{pr}} (y|H_1)/{\mathrm{pr}} (y|H_0)$ of the data and quantifies the extrinsic class separability (ECS). The difference between ICS and ECS measures the efficiency of data acquisition. We show that the extrinsic LR $\Lambda (y)$ is the expectation of the intrinsic LR $\Lambda_{I} (x)$, where the expectation is with respect to the posterior PDF ${\mathrm{pr}} (x|y, H_0)$ under $H_0$. *Main results*. We use two examples, one to clarify the new IO and the second to demonstrate its potential for real world applications. Specifically, we apply the new IO to spectral optimization in dual-energy CT projection domain material decomposition (pMD), for which SOMs are used to describe variability of basis material line integrals. The performance rank orders obtained by IO agree with physics predictions. *Significance.* The main computation in the new IO involves sampling from the posterior PDF ${\mathrm{pr}} (x|y, H_0)$, which are similar to (fully) Bayesian reconstruction. Thus our IO computation is amenable to standard techniques already familiar to CT researchers. The example of dual-energy pMD serves as a prototype for other spectral optimization problems, e.g., for photon counting CT or multi-energy CT with multi-layer detectors.

## 1. Introduction

Ideal observers (IOs) can quantify imaging hardware performance and compare hardware designs using task-specific image quality metrics (Barrett *et al* 1998). The decision variable of IOs is the likelihood ratio (LR) of the measured data, which depends on both the measurement model of the device and the object population characteristics. The noise mechanism of an imaging device is often well known from physics principles. The difficulty in computing the IO almost always lies in characterizing population variability.

One way to circumvent this difficulty is to remove or reduce the randomness in the population specification. This leads to the prevalent task designations such as (a) signal known exactly, background known exactly (SKE/BKE), where randomness in patients is completely removed, or (b) SKE, background known statistically (SKE/BKS), where the background (i.e. the normal cases) is random, but the signal is known and nonrandom. Practical implementation of SKE/BKS often employs simple random background models such as the lumpy model (Rolland and Barrett 1992), which is still far from real patient background. In addition, the computation of IO for SKE/BKS tasks involves special-purpose Markov chan Monte Carlo (MCMC) techniques (Kupinski *et al* 2003) that may not be well known in the image formation community. There is a need to further develop IO to make it suitable for clinical tasks with realistic or even free-form population variability, and to provide general-purpose tools for reliable and practical IO computation.

The importance of IO motivates the continued development of computational tools to estimate IO performance. Different methods have been proposed from different perspectives. By pre-computing an organ-based projection dataset and leveraging the linear data generation model in SPECT, He *et al* He *et al* (2008) were able to improve the MCMC sampling efficiency for an SKE/BKS task by on-the-fly recombination of organ projections to form the posterior samples. Seeking to further extend the domain of applicability of MCMC methods, Zhou *et al* (2023) used a generative adversarial network to sample realistic patient anatomical backgrounds for IO estimation in an SKE/BKS task. Additional efforts to make IO more accessible include Kupinski *et al* (2001), Zhou *et al* (2019, 2020), where the IO test statistic—the posterior LR—is obtained as outputs of a neural network via supervised training.

In this work we propose a new IO formulation for binary task performance evaluation. Not only is the new IO fully compatible with stochastic object models (SOMs) for clinically realistic patient population specification, an additional advantage is that estimation of IO performance can be accomplished by familiar, general-purpose computational tools commonly in use. A key difference between our formulation and the existing one is that whereas the existing approach treats objects as continuous-domain functions (of infinite dimension) and the imaging system as a continuous-to-discrete (CD), or an infinite-dimension to finite-dimension) mapping, we treat objects as finite-dimensional vectors and the imaging system a discrete-to-discrete (DD), a finite-dimension to finite-dimension) mapping. It is desirable to extend IO formulations to cover DD mappings as (a) they naturally occur in some ‘parametric imaging’ applications, and (b) even as approximate models, DD mappings are valuable for many data acquisition problems. These statements will be elaborated upon later in the paper.

The rest of the paper is organized as follows. We review the conventional IO for SKE/BKS tasks in section 2 to lay the groundwork. In section 3, we present the new IO formulation for DD mappings. We then use a toy example in section 4 to further clarify key concepts in the new IO; and in section 5 to showcase its practical use, we consider a dual-energy spectral optimization problem. In section 6 we discuss the (common) challenges for IO computation in high-dimensional settings and how our formulation aligns well with the latest development in generative AI that is capable of capturing population statistics. We conclude the paper in section 7 with potential topics for future works.

## 2. Background

A **linear** data acquisition system, denoted by $\mathcal{H}$, can be modeled as a CD mapping that makes an indirect observation of a population sample *x*:

\begin{align*} y = \mathcal{H } x\,+\,n\end{align*}

where *x* is the continuous object being imaged, $y \in R^{m}$ is the measured data, and $n \in R^{m}$ is the measurement noise.

For binary SKE/BKS tasks, the two underlying populations from which *x* arises are: ($H_0$) random background *x*_*b*_ only, and (*H*_1_) superposition of a known, non-random signal *x_f_* and the background *x_b_*. The task of the data acquisition system (1) is to determine from the measurement *y*, to which population or hypothesis the imaged object *x* belongs. The measurements corresponding to the two hypotheses can be written as

\begin{align*} &amp; H_0: \ y = {\mathcal{H}x_b } + n = b\,+\,n , \quad \qquad \qquad b \stackrel{\triangle}{ = }\mathcal{H} x_b\end{align*}

\begin{align*} &amp; H_1: \ y = {\mathcal{H} } \left(x_b + {x_f } \right) + n = b + \zeta + n, \quad \zeta \stackrel{\triangle}{ = } \mathcal{H} x_f,\end{align*}

where the background-only image $b \in R^{m}$ and the signal-only image $\zeta \in R^{m}$ are both defined in the data domain and are of finite dimension, unlike the background object *x_b_* or the signal object *x_f_*.

The IO uses the data LR as its decision variable, defined as $\Lambda(y) = {\mathrm{pr}} ( y |1)/ {\mathrm{pr}} (y |0)$, where ${\mathrm{pr}} (y|i) \equiv {\mathrm{pr}} (y|H_i)$ is the probability density of the data under hypothesis *H*_*i*_ (Kupinski *et al* 2003). The background data *b*, with distribution ${\mathrm{pr}} (b)$ induced from the random background object *x*_*b*_, make the marginal data distribution ${\mathrm{pr}} (y|i) = \int {\mathrm{d}} b \, {\mathrm{pr}} (y|b, i) {\mathrm{pr}} (b) $, $i = 0,1$, very often intractable. The following reformulation of $\Lambda (y)$ was proposed (Kupinski *et al* 2003) to facilitate IO computation:

\begin{align*} \Lambda \left(y\right) = \frac{ {\mathrm{pr}} \left(y|1\right)}{{\mathrm{pr}} \left(y|0\right)} &amp; = \frac{\int \, {\mathrm{d}} b \, {\mathrm{pr}} \left(y |b, 1\right) {\mathrm{pr}} \left(b \right) } {\int \, {\mathrm{d}} b^{^{\prime}} \, {\mathrm{pr}} \left(y |b^{^{\prime}}, 0 \right) {\mathrm{pr}} \left(b^{^{\prime}} \right) }\nonumber\\ &amp; = \frac{\int \, {\mathrm{d}} b \, \frac{{\mathrm{pr}} \left(y |b, 1\right) } { {{\mathrm{pr}} \left(y |b, 0\right) }} {\mathrm{pr}} \left(b \right) {{\mathrm{pr}} \left(y|b, 0\right) } } {\int \, {\mathrm{d}} b^{^{\prime}} \, {\mathrm{pr}} \left(y |b^{^{\prime}}, 0 \right) {\mathrm{pr}} \left(b^{^{\prime}} \right) } = \int \, {\mathrm{d}} b \, \boxed{\frac{{\mathrm{pr}} \left(y |b,1\right)}{{\mathrm{pr}} \left(y|b,0\right)}} \, {\mathrm{pr}} \left( b |y, 0\right) \nonumber\end{align*}

where the term in the box is recognized as the BKE LR, i.e. $ \Lambda_{\mathrm{BKE}} (y|b) = {\mathrm{pr}} (y|b,1) / {\mathrm{pr}} ( y|b, 0 ) $, which often has a closed-form expression derived from physics principles. The other component in (3), ${\mathrm{pr}} (b|y, 0)$, is the posterior distribution of the background *b* given data *y* under $H_0$. While still (often) intractable analytically, the posterior ${\mathrm{pr}} (b|y,0)$ is amenable to stochastic sampling using MCMC techniques. As a result, the LR $\Lambda (y)$ can be estimated using a sampled version of (3):

\begin{align*} \Lambda \left(y\right) \approx \frac{1}{J} \sum_{j = 1}^{J} {\frac{{\mathrm{pr}} \left(y|b_j,1 \right)}{{\mathrm{pr}} \left(y|b_j,0\right)} }, \qquad b_{j} \sim {\mathrm{pr}} \left(b|y, 0\right)\end{align*}

where $\Lambda (y)$ is estimated using the BKE LR $ \Lambda_{\mathrm{BKE}} (y|b_j)$ calculated using sample background (in the data domain) *b*_*j*_, $j = 1, \cdots, J$, drawn from the posterior distribution ${\mathrm{pr}} (b|y, 0)$. Here the notation ∼ means ‘to sample from’ (a distribution). This procedure (4) is then applied to many data samples *y* under each hypothesis, to obtain samples of LRs, from which summary metrics using the receiver operating characteristic (ROC) curve and the area under the curve (AUC) can be derived.

The CD-mapping treats SOMs as infinite-dimension random processes, which are difficult to directly work with or to obtain samples from. To circumvent the difficulty, the population statistics are characterized not in the object domain but through the CD-mapping in the data domain, i.e. through *b* and *ζ* in (2), for which probability density functions (PDFs) are assessed and used for LR computation.

For some applications, although the random objects are continuous spatial-domain random processes of an infinite dimension, the underlying random mechanism is of finite dimension. This is the case for the lumpy model (Rolland and Barrett 1992, Barrett and Myers 2004), pp 444, where randomness is controlled by the number of the lumps and the center location of each lump. Another example is the XCAT phantom family (He *et al* 2008), where anatomical variations can be controlled by parameter settings such as the organ size, shape, location, etc. In addition to the geometric parameters, another example can be found in the dual-energy CT application that we consider in section 5 where random material compositions or functional parameters constitute the SOMs. For these and possibly many other applications, it is natural to prescribe PDFs for the finite-dimensional random parameters and assess SOMs directly in the object space.

Data acquisition for such ‘parametric imaging’ applications can be considered as a DD mapping; any CD transformations, e.g., from the lump intensity profile to measurements, is absorbed into a ‘system’ matrix. IOs can be formulated to directly work with the finite dimensional PDFs of SOMs for hardware optimization.

## 3. Method

We inherit notation from section 2 wherever possible and introduce new ones related to the finite dimension SOMs. In contrast to the CD-mapping (2), the DD data model under each hypothesis can be written as

\begin{align*} &amp; H_0: \ y = \mathcal{H}_d \,x\,+\,n,\qquad x \sim p_0\left(x\right) \equiv {\mathrm{pr}} \left(x|0\right)\end{align*}

\begin{align*} &amp; H_1: \ y = \mathcal{H}_d \, x\,+\,n,\qquad x \sim p_1\left(x\right) \equiv {\mathrm{pr}} \left(x|1\right)\end{align*}

where $\mathcal{H}_d\in R^{m\times n_d} $ denotes the DD-mapping or the system matrix, $y \in R^{m}$ is the measurement, and the imaged object *x* is also of finite dimension, $x\in R^{n_d}$. In (5) we model object variability under $H_0$ and *H*_1_ using their individual PDFs $p_{0}(x ) \equiv {\mathrm{pr}} (x|0) $ and $p_{1} (x) \equiv {\mathrm{pr}} (x|1)$, while a similar statement can not be easily made for the random processes *x* in the CD-mapping (2). Unlike the CD mapping (1), the DD mapping $\mathcal{H}_d$ (5) should be understood as a possibly nonlinear operator; and the noise can be non-additive as well. In our spectral optimization example later, the measurements are nonlinear and follow the Poisson distribution (i.e. multiplicative noise).

In (5), the population PDFs $p_{0}(x)$, $p_{1} (x)$ can be quite general. They could be two PDFs from the same distribution family with different parameters, or from different distribution families. In fact, there is no need for $p_{0}(x)$ and $p_{1}(x)$ to have any parametric form. The requirement on them, from the IO computation point of view, is that given an object sample, both $p_{0}(x)$ and $p_{1}(x)$ can be computed in a black-box manner. Moreover, the notions of ‘signals’, or signal-present, signal-absent classes are not needed at this point. For some binary tasks, e.g., classification of benign and malignant lesions, enforcing the superposition of a ‘signal’ on top of a ‘background’ may appear unnatural. Nevertheless, such distinctions can be supplemented for specific instantiations of (5) if the binary task is indeed signal detection.

With the DD model (5), instead of (3), we rewrite $\Lambda (y)$ as the following to facilitate computation:

\begin{align*} \Lambda \left(y\right) = \frac{{\mathrm{pr}} \left(y|1\right)}{{\mathrm{pr}} \left(y|0\right)} &amp; = \frac{ \int \, {\mathrm{d}} x\, {\mathrm{pr}} \left(y|x, 1\right) {\mathrm{pr}} \left(x|1\right)}{ \int \, {\mathrm{d}} x^{^{\prime}}\, {\mathrm{pr}} \left(y|x^{^{\prime}}, 0\right) {\mathrm{pr}} \left(x^{^{\prime}}|0\right) }\end{align*}

where $ {\mathrm{pr}} (y|x,i )$ , $i = 0,1$, is the conditional PDF of the data *y* given $x \sim p_{i}(x) \equiv {\mathrm{pr}} (x|i)$. An overarching assumption for the new IO formulation is that *the conditional data distribution is the same under $H_0$ and H_1_*, i.e.,

\begin{align*} {\mathrm{pr}} \left(y|x,1\right) = {\mathrm{pr}} \left(y |x, 0\right) \stackrel{\triangle}{ = } p \left(y|x\right).\end{align*}

In other words, (7) means that the functional form of the conditional data distribution does not depend on class membership. This assumption makes sense as data acquisition itself is agnostic to class membership^3^3The assumption (7) is embedded in the data model such as (1), for which we do not state to which hypothesis *x* belongs. More specifically, in (2) the same data acquisition $\mathcal{H}$ is employed for the two populations: $H_0$ (normal) and *H*_1_ (abnormal) patients. It is not saying that data (co-)variance is the same under each hypothesis. Nor is it saying the measurement noise does not depend on signal intensity. Further clarifications can be found in numerical examples.. Then applying (7) to (6) and using the definition of the posterior distribution ${\mathrm{pr}} (x|y, 0)$:

\begin{align*} \Lambda \left(y\right) = \frac{ \int \, {\mathrm{d}} x\, \frac{{\mathrm{pr}} \left(x|1\right)}{{\mathrm{pr}} \left(x|0\right)} {\mathrm{pr}} \left(y|x, {0} \right) {\mathrm{pr}}\left(x|0\right) } { \int \, {\mathrm{d}} x^{^{\prime}}\, {\mathrm{pr}} \left(y|x^{^{\prime}}, 0\right) {\mathrm{pr}} \left(x^{^{\prime}}|0\right) } = \int {\mathrm{d}} x \boxed {\frac{{\mathrm{pr}} \left(x|1\right)}{{\mathrm{pr}} \left(x|0\right)} } \, {\mathrm{pr}} \left( x|y, 0\right).\end{align*}

The reformulation of $\Lambda (y) $ in (8) has a structure similar to (3). The boxed term in (8), taking the place of the BKE LR $\Lambda_{{\mathrm{BKE}}} (y)$ of (3), is recognized as the LR of the object *x*, followed by a posterior distribution ${\mathrm{pr}} (x|y, 0)$ that plays a similar role to ${\mathrm{pr}} (b|y, 0)$ in (8).

In the DD mapping, the imaged objects are finite dimensional random variables and can be characterized using PDFs. The LR of an object *x*,

\begin{align*} \frac{p_1\left(x\right)}{p_{0}\left(x\right)} \stackrel{\triangle}{ = } \Lambda_{I} \left(x\right)\end{align*}

will be called the intrinsic LR, $\Lambda_{I}(x)$, a quantity unique to the DD mapping. When $ \Lambda_{I} (x) $ is computed for a large number of samples *x* from both $p_{0}(x)$ and $p_{1}(x)$, we can derive summary measures such as the ROC curve and the AUC that quantify the intrinsic class separability (ICS), a property of the underlying population independent of data acquisition. By contrast, the data LR $\Lambda(y)$ (8) can be regarded as the extrinsic LR as it encodes the properties of a specific data acquisition method and the population statistics as well.

Using the notion of intrinsic and extrinsic LRs, the relationship (8) is saying that the extrinsic LR is the expected value of the intrinsic LR, where the expectation is taken with respect to ${\mathrm{pr}} (x |y, 0)$, the posterior probability under $H_0$ of the object *x* given the data *y*. A similar role of averaging in the CD formulation (3) is played by ${\mathrm{pr}} (b|y, 0)$. Here a notable difference—which affects IO computation—is that in the CD formulation the background *b* in $ {\mathrm{pr}} (b |y, 0) $ is in the data domain, whereas in the DD formulation, *x* in ${\mathrm{pr}} (x|y, 0)$ is the imaged object. If the DD mapping $\mathcal{H}_d$ in (5) is a forward projection operator, then ${\mathrm{pr}} (x|y, 0)$ is the familiar posterior distribution in Bayesian image reconstruction.

Just as in the CD case (4), we can estimate the extrinsic LR (8) using sample averaging:

\begin{align*} \hat{\Lambda} \left(y\right) \stackrel{\triangle}{ = } \frac{1}{J } \sum_{j} \frac{{\mathrm{pr}} \left(x_{j}|1\right)}{{\mathrm{pr}} \left(x_{j}|0\right)} = \frac{1}{J} \sum_{j} \Lambda_{I} \left(x_{j}\right) , \qquad x_{j} \sim {\mathrm{pr}} \left(x|y, 0\right).\end{align*}

The key to implementing (10) lies in generating the posterior samples $x_{j} \sim {\mathrm{pr}} (x|y, 0)$. As alluded to earlier, this issue is amenable to many Bayesian inference techniques, which will be discussed in section 5 with an application example.

Assuming the availability of a posterior sampler and a way to compute the intrinsic LR $\Lambda_{I}(x)$, the procedure to derive summary measures like the ROC curve or the AUC is the same as in the conventional IO. These summary measures quantify class separability as observed by an imaging device, which, to contrast with the ICS based on (9), is a notion of extrinsic class separability (ECS).

The ECS is dependent on both the imaging hardware and the population statistics, while the ICS is a property of the underlying populations exclusively and defines the fundamental task complexity. Data acquisition inevitably incurs irreversible information loss Barrett and Myers (2004), page 830, making the ECS inferior to the ICS. The ICS sets the performance upper bound for any imaging devices. If a task is designed with a reasonable complexity, i.e., with a reasonable ICS, the ECS can quantity task performance of an imaging device. A better ECS means that an imaging device is better at preserving class separability and achieves better task performance.

We mentioned that the LR (8) in the new IO is structurally similar to the LR (3) in the conventional IO. Notably, (8) is a generic expression for all binary tasks defined in (5). This brings out the first advantage of the new IO. That is, it is conceptually straight-forward as it considers a generic task upfront. A specific task definition, e.g., with either signal variability, or background variability, or both, then become a specific instantiation of the generic expression (8).

Below we apply the new IO to two instantiations of signal detection^4^4Our tasks can be designated as SKS/BKS, i.e. signal-known-statistically, background-known-statistically. Within the general framework of (5), this task specification leads to expressions that relate $p_1(x)$ to $p_0(x)$.. The first one is a toy problem, in which all PDFs have known expressions. We use it to elaborate on the conditional data assumption (7), the specifications of population PDFs $p_{0}(x)$, $p_{1}(x)$ for signal detection tasks, and in preparation for real applications, the computation of the intrinsic LR $\Lambda_{I} (x)$ (9) using MC techniques. The known PDFs provide the ground-truths for accuracy check. Building on the toy problem, we then consider a dual-energy spectral optimization problem for material decomposition. The emphasis there is on posterior sampling $x \sim {\mathrm{pr}} (x|y, 0)$ for computing the extrinsic LR (10).

For computation and illustration purposes, it can be more convenient to calculate log-LRs instead of LRs. Therefore we define $\lambda_{I} (x) \stackrel{\triangle}{ = }\log \Lambda_{I} (x)$ and for the sampled version, $\hat{\lambda}_{I} (x) \stackrel{\triangle}{ = } \log \hat{\Lambda}_{I} (x)$; and similarly, for the extrinsic LR, $\lambda (y) \stackrel{\triangle}{ = } \log \Lambda (y)$, $\hat{\lambda} (y) \stackrel{\triangle}{ = } \log \hat{\Lambda} (y)$, where $ \hat{\Lambda}(y) $ is the sample average version (10). These notation will be used in the following sections.

## 4. Application 1: a toy problem

We consider a one-pixel detector (*m* = 1) measuring a ‘line integral’ of a two-pixel ($n_d = 2$) object $x = [x_1, x_2]^t \in R^2$:

\begin{align*} y \stackrel{\triangle}{ = } h^t x\,+\,n = \left[ h_1 \ h_2 \right] \left[ \begin{array}{c} x_1 \\ x_2 \end{array} \right] + n,\end{align*}

where $n \sim \mathcal{N} (0, \sigma^2_d)$ is a uni-variate normal random variable modeling the measurement noise, and *x* may come from two distributions or classes, $p_{i}(x)$, for *i* = 0 or 1. Obviously, the data model (11) satisfies the conditional data assumption (7): the same conditional distribution

\begin{align*} {\mathrm{pr}} \left(y|x\right) = \mathcal{N} \left(h^t x , \sigma^2_d \right)\end{align*}

holds regardless of the class membership of *x*. We assume $p_{0}(x)$ is the background-only PDF given by a bivariate-normal (BVN) distribution:

\begin{align*} p_{0}\left(x\right) = \mathcal{N} \left(\mu_{0}, \Sigma_0 \right).\end{align*}

with known mean *µ*_0_ and (co-)variance matrix Σ_0_. The signal-present PDF $p_{1}(x)$ is derived from the superposition of a background-only object $x^{(0)} \sim p_{0}(x)$ and an independent, 2-pixel ‘signal’ *u* of a specific form. A signal-present object $x^{(1)}$ is written as

\begin{align*} x^{\left(1\right)} = x^{\left(0\right)} + u, \quad x^{\left(0\right)} \sim p_{0}\left(x\right), \quad u = \left[ \begin{array}{c} 0 \\ \mathrm{u} \end{array} \right], \ \mathrm{u} \sim p_{\delta} \left(\mathrm{u}\right) = \mathcal{N} \left( \nu, \sigma^2\right).\end{align*}

In other words, the signal *u* adds a uni-variate normal perturbation to the second pixel of the background-only image $x^{(0)}$. By construction, the signal-present PDF $p_{1}(x)$ is also a BVN given by:

\begin{align*} p_1 \left(x\right) = \mathcal{N} \left(\mu_1, \Sigma_1\right) \quad \mu_1 = \mu_{0} + \left[ \begin{array}{c} 0 \\ \nu \end{array} \right], \ \Sigma_1 = \Sigma_0 + \left[ \begin{array}{cc} 0 &amp; 0 \\ 0 &amp; \sigma^2 \end{array} \right].\end{align*}

Combining the data acquisition (11) with the SOM distributions (13)–(15), the measurement *y* is normally distributed under both hypotheses.

\begin{align*} H_{0} : \quad &amp; y \sim \mathcal{N} \left(h^t \mu_0 , \ h^t \Sigma_0 h + \sigma^2_d \right) ,\end{align*}

\begin{align*} H_{1} : \quad &amp; y \sim \mathcal{N} \left( h^t \mu_1 , \ h^t \Sigma_1 h + \sigma^2_d \right) .\end{align*}

Here due to the unequal co-variance matrix Σ_0_ and Σ_1_ in the SOMs (15), the measurement *y* has different variance under $H_0$ and *H*_1_. The conditional data assumption (7) does not rule out such data models.

The normal distributions make it easy to compute both (a) the intrinsic LRs $\Lambda_{I} (x) = p_1(x)/p_{0}(x)$, and (b) the extrinsic $\Lambda (y) = {\mathrm{pr}} (y|1)/{\mathrm{pr}}(y|0)$ using the analytic expressions. Such situations rarely happen in real applications. To prepare for them, next we consider MC methods to approximate these LRs when some prior knowledge can be assumed about the SOMs.

### 4.1. Intrinsic LRs and ICS

Here we assume (a) the background-only PDF $p_{0}(x)$ can be evaluated for each sample *x* in a black-box manner, and (b) the signal-present object is generated by the superposition of a background-only sample and a signal-only sample. The formulation (14) is such an example. With these assumptions, the PDF $p_{1}(x ) $ for class-1 samples is related to the background-only PDF $p_{0} (x)$ and the signal-only PDF as

\begin{align*} p_1 \left(x\right) &amp; = \int_{u \in U} \, {\mathrm{d}} u \, p_{0} \left( x - u\right) p_{\delta}\left(u\right)\end{align*}

where *U* denotes the sample space of the signals. Equation (17) says that $p_1(x)$ is a location-mixture version of $p_0(x)$. The intrinsic LR can be calculated using MC averaging as:

\begin{align*} \Lambda_{I} \left(x\right) { = } \frac{p_1 \left(x\right)}{p_0\left(x\right) } \stackrel{\left(17\right)}{ = } \int_{u \in U } \, \, {\mathrm{d}} u \, p_{\delta}\left(u\right) \, \frac{p_0\left( x - u\right)}{p_0\left(x\right)} \approx \frac{1}{S}\sum_{s = 1}^{S} \frac{p_0 \left(x - u_{s}\right)}{p_0\left(x\right) } = \hat{\Lambda}_{I} \left(x\right) , \quad u_{s} \sim p_{\delta}\left(u\right)\end{align*}

using sample signals *u*_*s*_, $s = 1, \cdots, S$, drawn from $p_{\delta} (u)$. Given a sample *x*, implementing (18) only requires (a) sampling from the signal PDF $p_{\delta} (\cdot)$, and (b) computing $p_{0}(x)$ for each *x*. Both are covered by our assumptions^5^5The sample *x* can be from either $H_0$, i.e., $x^{(0)} \sim p_0(x)$, or from *H*_1_ generated according to $x^{(1)} = x^{(0)} + u$, $x^{(0)} \sim p_0(x)$, $ u \sim p_{\delta } (u)$..

### 4.2. IO computation—ECS

By construction, the scalar measurement *y* under $H_0$ and *H*_1_ are both univariate normal as characterized in (16). It is straightforward to compute the extrinsic LR $\Lambda (y)$ which then leads to the IO summary measures.

An alternative is to estimate $\Lambda (y)$ using the posterior samples ${\mathrm{pr}} (x|y,0)$ (10), which could be combined with the MC approach (18) for estimating the intrinsic LR:

\begin{align*} \hat{\Lambda} \left(y\right) &amp; = \frac{1}{J} \sum_{j} \frac{p_1\left(x_{j}\right)} {p_0 \left(x_j\right)}, \quad x_{j} \sim {\mathrm{pr}} \left(x|y, 0 \right) \nonumber \\ &amp; \approx \frac{1}{J S} \sum_{j,s} \frac{p_0 \left(x_j - u_{s}\right)}{p_0\left(x_j\right)} \stackrel{\triangle}{ = } \hat{\hat {\Lambda}} \left(y\right), \quad u_{s} \sim p_{\delta}\left(u\right), \quad x_{j} \sim p\left(x|y, 0 \right).\end{align*}

For the toy example, the posterior ${\mathrm{pr}} ( x|y, 0)$ can be computed using ${\mathrm{pr}} ( x|y, 0) = C {\mathrm{pr}} (y|x )p_0 (x)$, where *C* is the normalization constant $p(y|0)$. From (12) and (13), both ${\mathrm{pr}} (y|x)$ and $p_0 (x)$ follow the normal distribution; the posterior ${\mathrm{pr}} (x|y, 0)$, via completion of squares, can be shown to be a BVN as well:

\begin{align*} {\mathrm{pr}} \left(x |y, 0\right) = \mathcal{N} \left(\mu_{|y}, \Sigma_{|y} \right), \quad \Sigma_{|y} \ = \left( h \, h^t / \sigma^2_{y,0} + \Sigma^{-1}_{0} \right) ^{-1} , \ \mu_{|y} = \Sigma_{|y} \left( \Sigma^{-1}_{0} \mu_0 + h\, y/ {\sigma^2_{y,0}} \right)\end{align*}

and $\sigma^2_{y,0} = h^t \Sigma_0 h + \sigma^2_d$ is the variance of the data *y* under $H_0$ (cf (16*a*)). Note that in (20), $h^t = [h_1, h_2] $, and $h h^t$ is a rank-1 $2 \times 2$ matrix.

For real applications, it rarely happens that there is a closed-form expression for the posterior PDF ${\mathrm{pr}} (x |y , 0)$ to sample from. Advanced Bayesian techniques to sample from a posterior distribution without having a full expression is needed. This topic will be discussed in our second application example. Before that, here we illustrate with a numerical example the materials we have so far.

### 4.3. Numerical results

#### 4.3.1. Data generation and ICS

**Figure 1.**
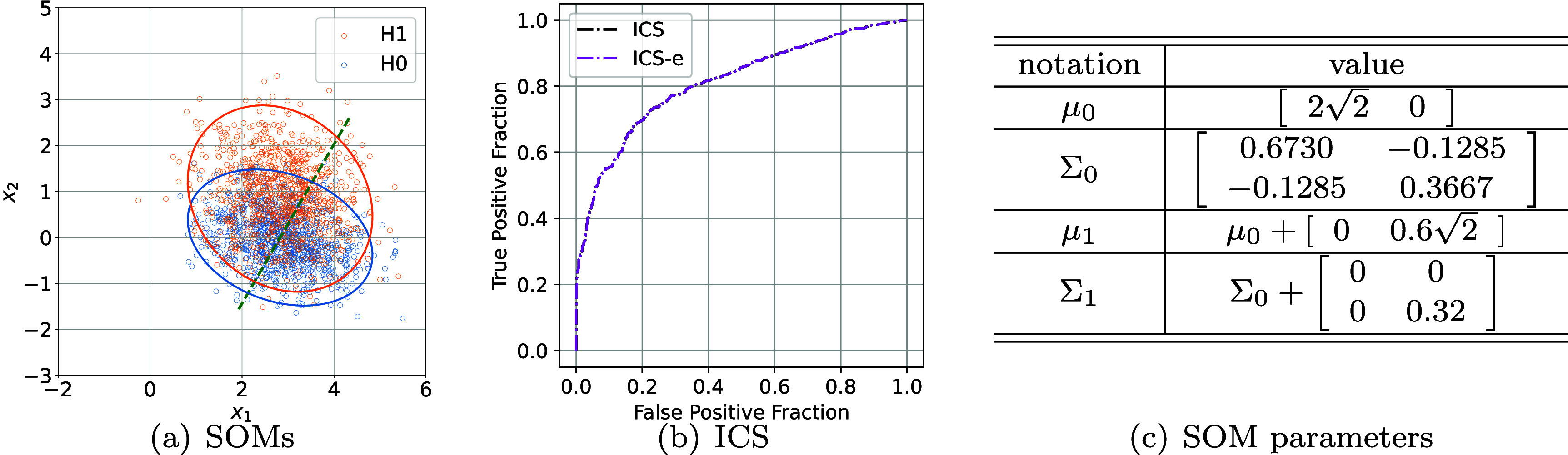
SOMs and intrinsic class separability (ICS). (a) 1000 samples each from $H_0$ and *H*_1_ and their respective 95% confidence ellipse. The dashed green line shows an example 1-pixel detector. (b) Two ROC curves, constructed using scikit-learn’s roc_curve function, for ICS obtained using the ground truth (gt) $\lambda_{I} (x)$ and the estimated $\hat{\lambda}_{I}(x)$ using (18). The ROC curves overlap and can not be distinguished. The estimated AUC is 0.8129 ± 0.0095. (c) SOM parameters for $H_0$ and *H*_1_.

We generated bi-variate normal SOM samples, using (13) for $H_0$ and (15) for *H*_1_. Shown in figure 1(a) are 1000 samples for each class. The unequal covariance matrices, Σ_0_ and Σ_1_, originated from the signal model (14), can be seen from the 95% confidence ellipse (CE) for both $H_0$ and *H*_1_.

**Figure 2.**
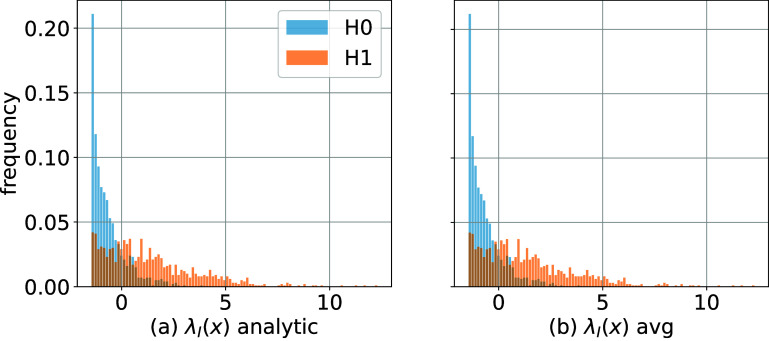
(a) Histograms of 1000 samples of the intrinsic $\lambda_{I} (x)$ under $H_0$, *H*_1_ calculated using analytic PDFs. (b) Same as (a) but estimated using MC averaging (18). (a) and (b) share the same vertical axis.

We calculated the log-LRs $ \lambda_{I} (x) = \log \Lambda_{I} (x) = \log [ p_1(x) / p_{0}(x) ]$ using (i) the analytic PDF expressions for $p_{0} (x)$, $p_{1}(x)$ from (13) and (15); and (ii) using the MC approximation $\hat{\lambda}_{I} = \log \hat{\Lambda}_{I}(x)$ (18) with the analytic expression of $p_{0}(x)$ and 1000 signal samples *u*_*s*_ for each *x*. The histograms of the log-LRs for (i) and (ii) are shown in figure 2. It can be seen that the histograms are almost identical, confirming that the difference between the two methods is small.

Lastly, the log-LRs $\lambda_{I} (x)$ of figure 2 were analyzed using ROC methodology. Specifically, we used scikit-learn’s roc_curve function to estimate the ROC from samples of $\lambda_{I}(x)$ shown in figures 2(a) and (b). The summary ROC curves from them completely overlap (figure 1(b)); and both yield the same AUC of 0.8129 ± 0.0095.

#### 4.3.2. IO performance—Extrinsic class separability

We parameterize the scalar detector as $ h^t = [h_1, h_2] = [\cos\theta, \sin\theta ]$, where $\theta \in [0, 2 \pi]$; the detector noise standard deviation was $\sigma_\mathrm{d} = 0.3$ (cf (12)). As *θ* varies, the detector measures different combinations of *x*_1_ and *x*_2_. Some orientations are better at preserving the separability between $H_0$ and *H*_1_ samples, leading to better ECS. However, due to the reduced dimension of the measurement (2 inputs, but 1 measurement) and the detector noise (*σ*_*d*_), we expect lower class separability from the data *y* than from the original samples *x*. In other words, we expect ECS $\unicode{x2A7D}$ ICS.

**Figure 3.**
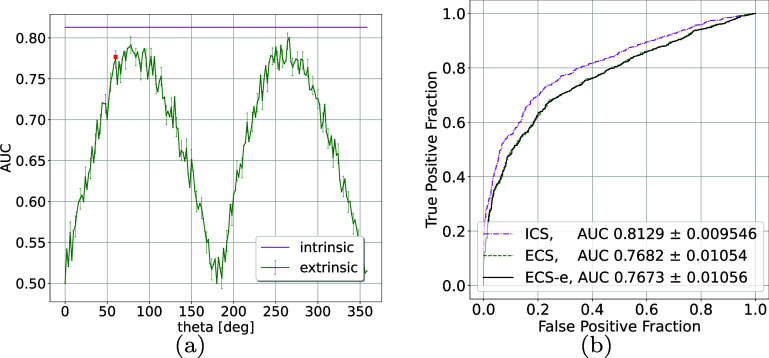
Extrinsic class separability calculated using $\lambda (y) = \log ({\mathrm{pr}} (y|1)/{\mathrm{pr}} (y|0))$. (a) The extrinsic AUCs (green curve) change for different measurement angle *θ*, while the ICS (magenta) remains the same. The red dot marks the position of the detector at $\theta = 60^{\circ}$, the dashed green line in figure 1. (b) The extrinsic ROC curves, calculated using analytic $\lambda (y)$ (16) (with label ECS) and using MC $\hat{\hat{\lambda}}(y)$ (19) (with label ECS-e) for detector angle $\theta = $ 60^∘^. We also replotted the intrinsic ROC from figure 1 for comparison.

The ECS was calculated using log-LRs $\lambda (y) $ from (16) for each *θ*. For example, when *θ* = 0, the detector is parallel to the *x*_1_ axis; the measured values—mostly *x*_1_ plus noise—for $H_0$ and *H*_1_ almost completely overlap. As shown in figure 3, the maximum ECS is obtained when the detector is nearly vertical $\theta \approx 90^{\circ}$. We also observe that ECS is lower than the (constant) ICS for all *θ*, due to the measurement noise and dimension reduction.

**Figure 4.**
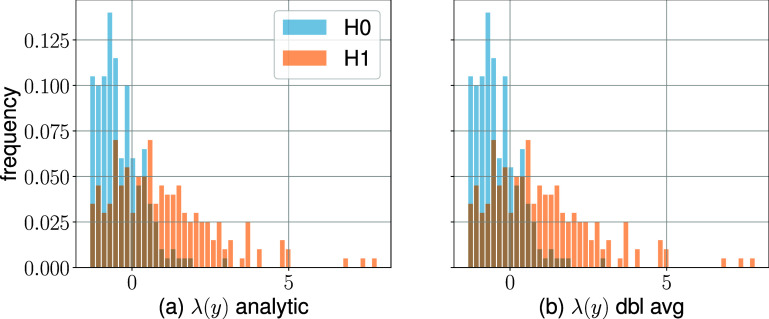
The extrinsic log-likelihood ratio $\lambda (y)$ for detector position at $\theta = 60^\circ$. Histograms of (a) the ground truth $\lambda (y)$ and (b) the estimated $ \hat{\hat{\lambda}} (y)$. Both (a) and (b) share the same vertical axis.

For $\theta = 60^\circ$ (the dashed green line in figure 1), we also estimated the extrinsic log-LR $\lambda (y) $ using the MC approximation (19) with posterior samples obtained from the closed-from expression (20). The histograms of $\lambda (y)$ and $\hat{\lambda} (y)$, shown in figure 4, are again almost identical. Similar to the intrinsic case, the log-LRs $\lambda (y)$, $\hat{\lambda}(y)$ were analyzed using ROC methodology. The summary ROC curves, labeled with ECS (from $\lambda(y)$, (16)) and ECS-e (from $\hat{\hat{\lambda}}(y)$, (19)) in figure 3(b), overlap and yield almost identical extrinsic AUCs. For comparison, we also re-plotted the intrinsic ROC curve from figure 1, which is above the extrinsic ROC for all false positive fraction (FPF) values.

## 5. Application 2: dual-energy CT

We turn to a realistic application, namely, a dual-energy spectral optimization problem. One way to implement dual-energy CT is to acquire two sets of CT data at two kVp settings, e.g., 80 kVp and 140 kVp. If such acquisitions are spatially aligned, i.e., the same line-integrals are acquired twice at different kVs, then projection domain material decomposition (pMD) (Xu and Noo 2024) can be applied to decompose the two measured line integrals into two basis material (BM) thickness, e.g., water and iodine, which can then be reconstructed to form two BM maps. An important issue in dual-energy CT is spectral optimization, as different combinations of low and high kVp settings greatly affect the accuracy and precision of the material maps.

The line-by-line pMD is the inverse problem of a forward model, in which an imaging system acquires 2 (dual-energy) measurements from a 2-pixel image, i.e., two BM thicknesses. The combinations of BM thicknesses for different line integrals in a standard patient can be treated as a SOM. A spectral optimization problem can be set up for the binary task of detecting the presence of a weak, possibly random, signal corresponding to an increase in iodine concentration.

Although still a 2-pixel problem, two issues render the data distribution ${\mathrm{pr}} (y |1)$, ${\mathrm{pr}} (y|0)$, and the posterior distribution $ {\mathrm{pr}} (x|y,0)$ intractable: (i) a more sophisticated SOM for the BM thickness combinations in a patient or a phantom, and (ii) the nonlinear forward model. Direct posterior sampling as in (20) is infeasible. Additional techniques to (approximately) sample from ${\mathrm{pr}} (x|y, 0)$ are required.

### 5.1. Data acquisition model

As mentioned earlier, one way to implement dual-energy CT is to acquire spatially aligned measurements, one at a low kVp, the other at a high kVp. In material decomposition, the measured transmission data *y*_*l*_ and *y*_*h*_ are (conditionally) independent Poisson random variables, with means $\bar{Y}_{l}$, $\bar{Y}_{h}$ modeled as

\begin{align*} \bar{Y}_{i} \left(x_1, x_2\right) &amp; = \sum_{b} N_{i}\left(E_{b}\right) \mathrm{e}^{- \left[ \mu_1 \left(E_b\right) x_1 + \mu_2 \left(E_{b} \right) x_2 \right]}\end{align*}

where $N_{i} (\cdot ) $, $ i = \{l, h \}$ denotes the energy spectrum combining effects of the low (or high) tube spectrum, the bowtie filter, and the detector energy response; $\mu_{1} (E_{b})$ and $\mu_{2} (E_{b})$ are the BM linear attenuation coefficients at energy *E*_*b*_, with discrete energy bin index *b* ranging from 5 keV to 140 keV, and $x = (x_{1}, x_{2}) \in R^{2}$ is a 2-pixel image representing a 2-tuple of BM thicknesses. In this work, we use water and iodine as our BMs, and identify *x*_1_ with water thickness, *x*_2_ with iodine thickness. Combining (21) with the Poisson noise model, the conditional data distribution can be written as

\begin{align*} {\mathrm{pr}} \left(y_{l}, y_{h} |x_{1}, x_{2}\right) &amp; = \prod_{i = \left\{l,h \right\} }{{\mathrm{Pois}}} \left( y_{i} ; \bar{Y}_{i} \left(x_{1}, x_{2}\right) \right)\end{align*}

where ${{\mathrm{Pois}}} ( a ; b)$ denotes a Poisson random variable *a* with mean *b*.

We will specify the stochastic models, $p_{0} (x)$, $p_{1}(x)$, shortly. But it should be pointed out now that the conditional data assumption (7) still holds: the conditional distribution (22) works for both $ x \in p_{1} (x)$ and $x \in p_{0} (x)$. For Poisson distribution the noise variance depends on the mean, therefore this is an example showing that the conditional data assumption (7) is not saying that the data noise is independent of image intensity. Data noise can certainly depend on image intensity, but it depends on image intensity in the same manner regardless of its class membership.

### 5.2. Stochastic object models

A dual-energy CT system should work well for various 2-tuple BM thicknesses that exist in a patient. Such normal ensembles of BM thicknesses will be characterized by the signal-absent PDF $p_{0} (x)$. In our numerical example, we calculate pairs of (water, iodine) thickness for all lines passing through an XCAT phantom (Segars *et al* 2018), and fit the resulting 2D histogram using a Gaussian mixture model (GMM):

\begin{align*} p_{0}\left(x\right) &amp; = \sum_{c = 1}^{C} \pi_{c} \mathcal{N} \left( \mu_{c}, \Sigma_{c}\right)\end{align*}

where $ \pi_{c} \unicode{x2A7E} 0$, $\sum_{c} \pi_{c} = 1 $, are the component weights, and *µ*_*c*_, $\Sigma_{c}$ are the BVN parameters for each component, $c = 1, \cdots, C$, and *C* is the number of mixture components.

A dual-energy CT system should be sensitive to weak increase of contrast materials. We formulate this task by deriving a signal model from the forward projection of an iodine disk insert of a certain radius and intensity, but at a random location within the object.

For each line $\ell$ passing through a disk of radius *R*, the chord length is given by $\delta = 2 \sqrt{R^2 - \varrho^2}$, where *ϱ* is the radial distance of the line $\ell$ from the center of the disk. One way to model the pathlength variation through the disk is to treat *ϱ* as a uniformly distributed random variable within $ [-R, R]$, which then induce a density function for the signal pathlength *δ*. The signal-present SOM can be modeled as

\begin{align*} x^{\left(1\right)} = x^{\left(0\right)} + u , \quad x^{\left(0\right)} \sim p_{0} \left(x\right) \quad u = \left[ \begin{array}{c} 0 \\ \delta \end{array} \right], \quad \delta = 2 \sqrt{R^2 -\varrho^2}, \quad \varrho \sim \mbox{Unif} \left( \left[-R, R\right] \right).\end{align*}

It may be cumbersome to derive the PDF $p_{1} (x)$ for $x^{(1)}$ in (24), but it is easy to sample the signal *u* so that the MC approach (18) can still be applied to calculate the intrinsic LR $\Lambda_{I} (x)$ and the ICS.

### 5.3. Sampling from the posterior distribution

To calculate the IO decision variables using either (10) or (19), we need to sample from the posterior distribution, ${\mathrm{pr}} (x |y, 0)$, expressed as

\begin{align*} {\mathrm{pr}} \left(x|y, 0 \right) &amp; = \frac{{\mathrm{pr}} \left(y|x, 0\right) p_{0} \left(x\right)}{{\mathrm{pr}} \left(y|0\right)} \stackrel{\left(7\right)}{ = } \frac{p \left(y|x \right) p_{0} \left(x\right)}{{\mathrm{pr}} \left(y|0\right)}\end{align*}

where ${\mathrm{pr}} (y |0) = \int_{x} \,{\mathrm{d}} x \, p (y |x ) p_{0}(x) $ is the partition function or the marginal distribution of the measurement *y* under $H_0$. Unlike the toy example, the more sophisticated SOM $p_{0}( x)$ and the nonlinear data model (21) and (22) make the partition function ${\mathrm{pr}} ( y |0) $ intractable, and it is infeasible to sample directly from ${\mathrm{pr}} (x|y, 0)$.

Sampling from posterior distributions is an active topic in both machine learning and statistics. Many techniques have been proposed to either (a) estimate a parametric form for the posterior distribution or (b) apply MCMC to sample from it in an asymptotic manner. As the partition function ${\mathrm{pr}} (y|0)$ in (25) is inaccessible, these methods rely on the non-normalized version of the posterior (25) or on the score function $\nabla_{x} \log {\mathrm{pr}} (x|y, 0)$. Define

\begin{align*} \phi \left(x\right) \stackrel{\triangle}{ = } \left\{ p \left(y |x \right) p_{0} \left(x\right) \right\}\end{align*}

as the non-normalized posterior, (which also is the joint distribution ${\mathrm{pr}} (x, y |0)$), the score function of ${\mathrm{pr}} (x|y, 0)$ is

\begin{align*} \nabla_{x} \log p \left(x|y, 0\right) = \nabla_{x} \log \phi \left(x\right) = \nabla_{x} \log p\left(y|x\right) + \nabla_{x} \log p_{0}\left(x\right).\end{align*}

Note that the score function does not depend on the partition function ${\mathrm{pr}} (y|0)$, and can be evaluated conveniently using the non-normalized posterior $\phi (x)$.

Below we consider an approximate method, namely, the Laplace approximation, that samples from the posterior ${\mathrm{pr}} (x|y,0)$ via $\log \phi (x)$ and $ \nabla_{x}\log \phi (x)$. This method is simple yet widely used in machine learning (Azevedo-Filho and Shachter 1994), Ritter *et al* 2018). It approximates the posterior ${\mathrm{pr}}(x|y, 0)$ using a multi-variate normal (MVN) distribution, with the mean and covariance matrix estimated as follows.

Let $\hat{x}_{o}$ be the mode of the (log-) posterior from (26),

\begin{align*} \hat{x}_{o} &amp; \stackrel{\triangle}{ = } \arg\max_{x} \log \phi\left(x\right).\end{align*}

Then denote by $\hat{\Sigma}_{o}$ the negative Hessian matrix of the log-posterior evaluated at $\hat{x}_o$:

\begin{align*} \hat{\Sigma}_{o} \stackrel{\triangle}{ = } - \left[\left. \frac{\partial^2 \log \phi\left(x\right)}{\partial x_{i}x_{j}} \right|_{\hat{x}_{o}} \right].\end{align*}

The Laplace approximation amounts to

\begin{align*} {\mathrm{pr}} \left(x|y,0\right) \approx \mathcal{N} \left(\hat{x}_{o}, \hat{\Sigma}_{o}\right).\end{align*}

Note that in (28)–(30), both $\hat{x}_{o}$ and $\hat{\Sigma}_{o}$ are functions of the measurement *y* as in $\hat{x} \equiv \hat{x}_o( y)$, $ \hat{\Sigma} \equiv \hat{\Sigma}_{o}(y)$; this dependency is sometimes omitted without confusion.

In terms of computation, finding the mode $\hat{x}_{o}$ (28) is the same as solving a maximum *a posteriori* (MAP) problem. The objective $\log \phi(x)$, from (26) and (27), consists of the Poisson likelihood of the data (22) and the prior GMM $p_{0}(x)$ (23). We used scipy.optimize’s implementation of the truncated Newton method to estimate $\hat{x}_{o}$. Once $\hat{x}_{o}$ is obtained, evaluating the 2×2 negative Hessian $\hat{\Sigma}_{o}$ is easy. For high dimensional problems, e.g., one that involves a MAP *image* reconstruction to estimate $\hat{x}_{o}$, evaluating the full matrix $\hat{\Sigma}_{o}$ (29) can be problematic. Alternative strategies that sample from (30) without computing the Hessian may be needed (Orieux *et al* 2012, Gilavert *et al* 2014). It is worth reiterating that $\log\phi(x)$ of (28) is exactly the same objective as in Bayesian reconstruction. This connection should make IO computation more appealing and accessible to the image reconstruction community^6^6The term ‘Bayesian reconstruction’ can be ambiguous, as it sometimes refers to MAP reconstruction that generates a point estimate to capture the mode as in (28). Posterior sampling is akin to ‘fully’ Bayesian reconstruction, where the goal is to characterize the uncertainty in the reconstructed images..

### 5.4. Numerical results

Again we use a concrete numerical example to summarize the material in this section. We present our results on IO computation incorporating realistic SOMs for three dual-energy data acquisition schemes.

#### 5.4.1. Stochastic object models and ICS

**Figure 5.**
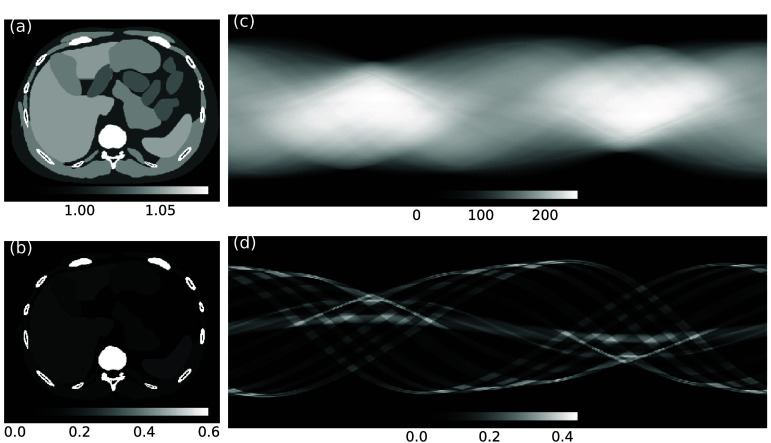
(a) Water density in g ml^−1^ and (b) iodine density in mg ml^−1^ in the XCAT phantom. (c) and (d): Fan-beam projection of water and iodine density map, both in units of g ml^−1^ × mm.

We created noise-free XCAT phantoms at two mono-energetic energies of 60 keV and 100 keV. The linear attenuation coefficient was decomposed into water and iodine density images (figures 5(a) and (b)) using an image domain material decomposition method (direct matrix inversion) (Taguchi *et al* 2007, Szczykutowicz and Chen 2010, Kappler *et al* 2013). The material density maps were then forward projected in a fanbeam geometry (source to iso-center distance 595.0 mm, source to detector distance 1085.6 mm) (Xu and Noo 2024) to generate material thickness line-integrals (figures 5(c) and (d)). The projection data consisted of 736 channels and 1152 views for a 360${}^\circ$ acquisition.

**Figure 6.**
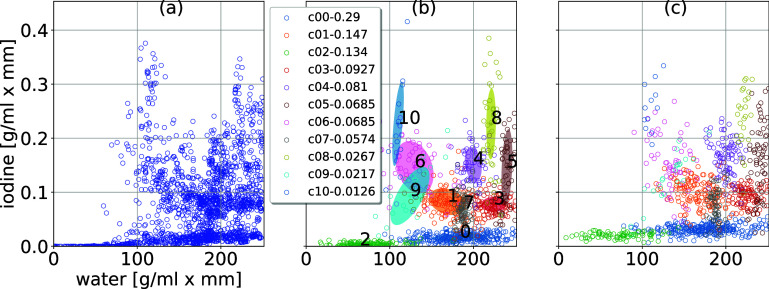
(a) Scatter plots of (water, iodine) combinations in the XCAT phantom. (b) The SOM $p_0(x)$ for $H_0$, obtained by GMM fitting for the scatter plots in (a). The legend shows the mixture weight, sorted from high to low. (c) The SOM $p_1 (x)$ for *H*_1_ obtained by a random vertical shift, representing a small iodine increment as discussed in section 5.2.

The scatter plot in figure 6(a) corresponds to (water, iodine) thickness pairs for the line integral in figures 5(c) and (d). These BM thickness pairs were then used to fit a GMM (23) with 11 mixture components. The component weight *π*_*c*_ (sorted from high to low), center *µ*_*c*_, and covariance matrix $\Sigma_{c}$ (illustrated by the 95% CE) are shown in figure 6(b). The fitted GMM is the SOM $p_{0}(x)$ for $H_0$. As described by (24), we created the SOM for *H*_1_ using a random signal that models a 20 mm diameter ($2R = 20$ mm in (24)) cylindrical insert with 1 mg/ml iodine solution that may appear anywhere within the XCAT phantom. Samples from the signal-present SOM under *H*_1_ are shown in figure 6(c). Comparing figures 6(b) and (c), it is noticeable that there is an overall vertical shift in figure 6(c). As the amount of shift is random, an analytic expression for $p_{1}(x)$ might be cumbersome.

**Figure 7.**
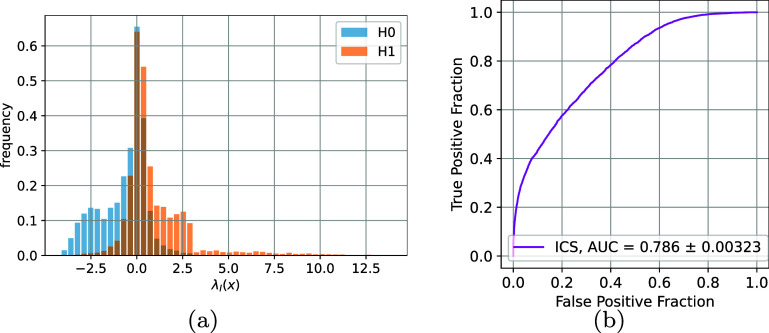
(a) Intrinsic log-likelihood ratios, $\lambda_{I} (x ) = \log( { p_{1} (x) }/ {p_{0} (x) }) $ for both *H*_1_ and $H_0$. (b) The ROC curve obtained using the ratings in (a). The summary ICS AUC is $ 0.786 \pm 0.00323$.

We calculated the intrinsic log-LRs $\lambda_{I} (x) $ using 10 000 samples each for $H_0$ and *H*_1_ (20 K in total) for the MC approximation (18). The histograms of $\lambda_{I} (x)$ and the summary ROC curve are shown in figure 7.

The SOM samples in figures 6(b) and (c) indicate our SOMs under $H_0$ and *H*_1_ have quite substantial overlap. This is reflected in the overlapping histograms in figure 7 and the moderate intrinsic AUC of 0.786. The task of hardware optimization is to maintain the class separability and achieve an extrinsic AUC as close as possible to this value.

#### 5.4.2. Data acquisition

**Figure 8.**
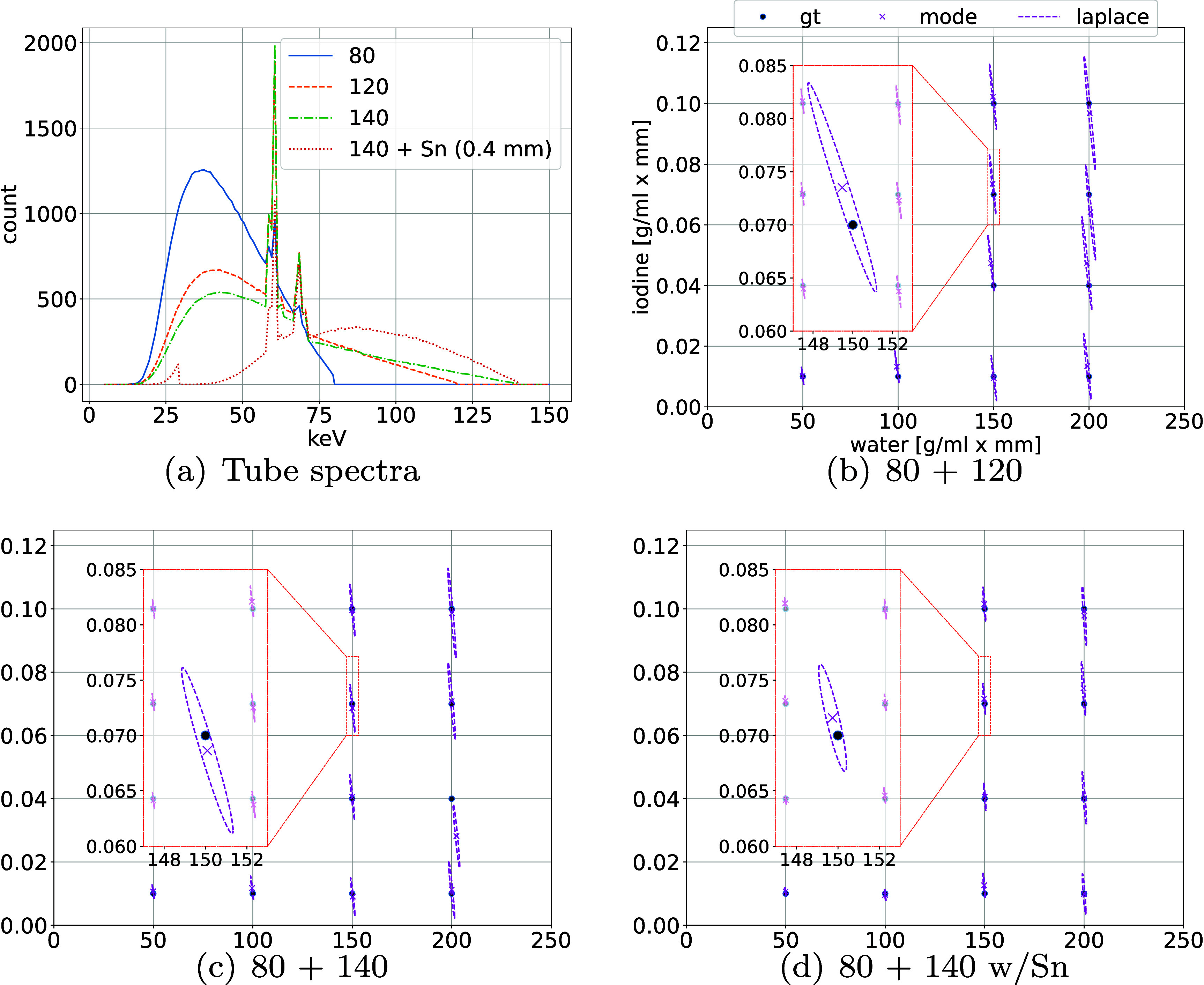
(a) Air-scan balanced tube spectra. (b)–(d) Laplace approximation for different spectral combinations. The covariance matrix from the Laplace approximation (30) is shown using the 95% CE. The insets show the same zoomed-in region for all three cases in (b)–(d). The setting of 80 + 140 w/Sn has the smallest uncertainty.

For the 20 K pairs of (water, iodine) samples, we generated noisy transmission data $(y_{l}, y_{h})$ using the data model (21) and (22) for three dual-energy acquisition strategies. They all share the same low voltage of (a) 80 kVp, but with different high voltages at (b) 120 kVp, (c) 140 kVp, and (d) 140 kVp with an additional 0.4 mm Sn filter. These tube spectra were obtained using the Siemens in-house simulation package ‘drasim’ (Fung *et al* 2011). We assume that the tube currents are adjusted to achieve the same air scan intensity of $2\times 10^6$ for all tube voltage settings. These air-scan balanced tube spectra are shown in figure 8(a). The effective energies of the spectra are, 44.5 keV, 55.9 keV, 60.8 keV, 85.9 keV, for the spectrum setting (a)–(d) respectively.

#### 5.4.3. Posterior sampling

We evaluated the performance of the Laplace approximation (30) for posterior sampling from ${\mathrm{pr}} (x|y,0)$ on a 4×4 grid of (water, iodine) thicknesses that span the range of variations in the SOMs (cf figures 6(b) and (c)). The results are summarized in figures 8(b)–(d). At each of the 4×4 grid points, the Laplace approximation is denoted by the 95% CE representing the covariance (29) with the mode $\hat{x}_{o}$ (28) marked by ‘texttimes’. For comparison, the gt is indicated with a solid circle ($\bullet$).

For each spectral combination, we notice that the size of the 95% CE increases when either the water thickness or the iodine thickness increases. The higher uncertainty in the posterior samples is due to the higher noise in the measurement $(y_l, y_h)$ caused by the larger BM thicknesses. When comparing different spectral settings, we notice that qualitatively, there is higher uncertainty for the 80 + 120 combination (figure 8(b)), and lower uncertainty for 80 + 140 (figure 8(c)), and even lower with the addition of the Sn filter (figure 8(d)). These observations all agree with the physics intuition that larger spectral separation leads to better conditioning and less uncertainty in material decomposition.

#### 5.4.4. IO performance—Extrinsic class separability

We provide in algorithm 1 the pseudo-code for computing the extrinsic log-likelihood $\lambda (y)$^7^7IO implementations for the toy example and the dual-energy CT example are available at https://tinyurl.com/3ftb2b4v... For our setup of dual-energy spectral optimization, the computation-intensive part is on line 4, sampling from the posterior distribution ${\mathrm{pr}} (x|y, 0)$ as each one is a pMD for one data sample $y = (y_l, y_h)$. Line 8 abstracts the procedure that computes the ROC curve and the AUC from the log-LRs $\lambda(y)$. Many tools, e.g. Matlab ROC function and free resources (2008), are available for this purpose. In this work, we used the roc functions in scikit-learn.

**Table:** 

**Algorithm 1.** Calculating the extrinsic $\lambda (y)$ and IO for dual-energy acquisition.
**Input:** The transmission data, $ y^{(0)}_{i} = (y^{(0)}_{l,i}, y^{(0)}_{h,i})\, y^{(1)}_{j} = (y^{(1)}_{l,j}, y^{(1)}_{h,j})$, for $H_0$ and *H*_1_ samples; GMM parameters for evaluating $p_{0}(x)$ (23);
**Output:** $\lambda^{(0)}_{i} \stackrel{\triangle}{ = } \lambda(y^{(0)}_{i}) $, $\lambda^{(1)}_{j} \stackrel{\triangle}{ = } \lambda(y^{(1)}_{j}) $, $\forall i, j $, the ROC curve, and AUC.
1 **for** *class* $c = 0, 1 $ **do**
2 $i_C \gets $ number of data samples
3 **for** *data sample* $i = 1, \cdots, i_C $ **do**
4 $x_{t} \sim {\mathrm{pr}} (x |y^{(c)}_{i}, 0) $ using (30), $t = 1, \cdots, T$
5 sample *u*_*s*_ using (24), $s = 1,\cdots, S$
6 $\Lambda = \sum_{t,s} p_{0} (x_{t} - u_{s}) /p_{0} (x_{t}) $ /* cf (19) */
/* normalize by sample size */
7 $ \lambda^{(c)}_{i} = \log \big (\Lambda / (T \cdot S) )$,
8 FPF, TPF, AUC $\gets$ ROC analysis $ (\lambda^{(0)}_{i}, \lambda^{(1)}_{j}, \forall i,j) $

**Figure 9.**
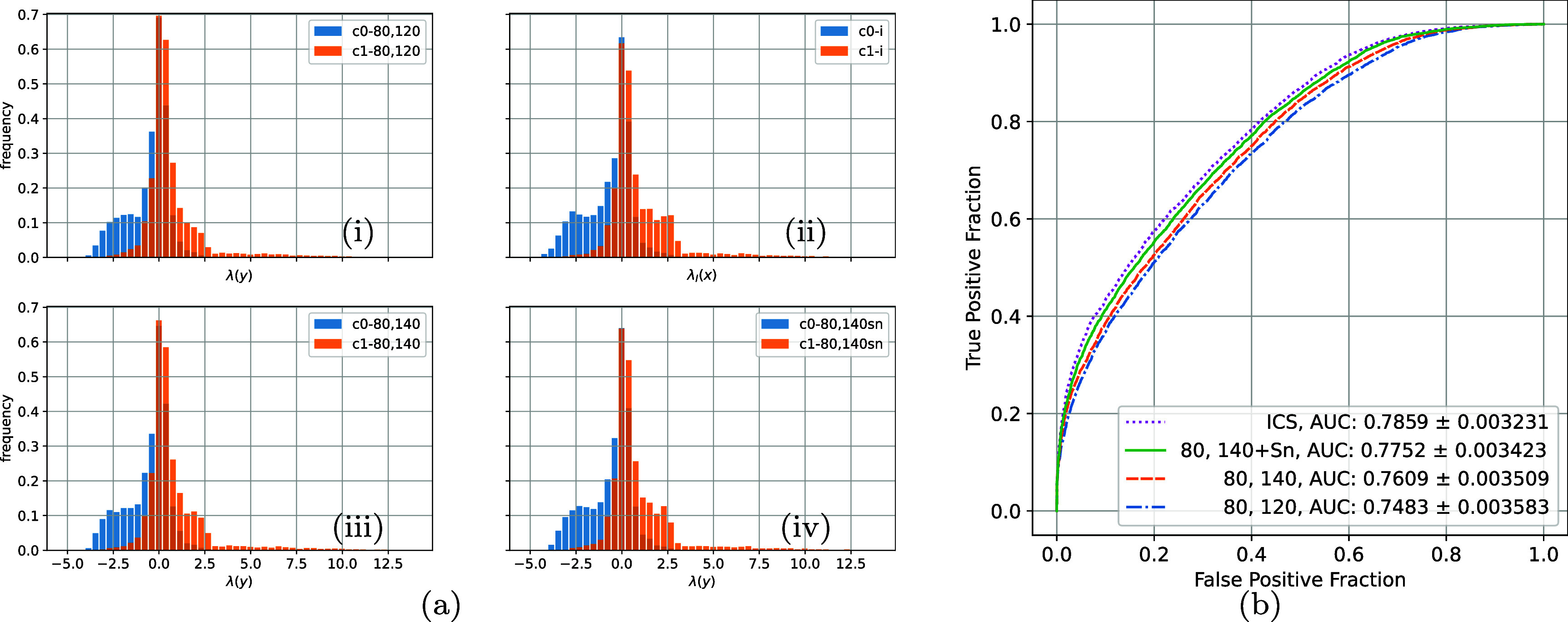
(a) The histograms of the intrinsic and the extrinsic log-likelihood ratios calculated for the three DE spectral combinations: (i) 80 + 120, (ii) intrinsic, (iii) 80 + 140, (iv) 80 + 140 w/ Sn. (i)–(iv) share both the *x* and *y* axes. (b) The summary ROC curves and the AUCs from the log-likelihood ratios in (a). The intrinsic log-likelihood ratios and ROC curve are replotted from figure 7 for comparison.

We ran algorithm 1 for each of the three DE strategies. The histogram of data log-LRs $\lambda(y)$ for $H_0$, *H*_1_, and the summary ROC curves and AUCs are shown in figure 9. For ease of comparison, we also replotted the intrinsic $\lambda_{I}(x)$ and the summary ROC curves from figure 7.

Consistent with the uncertainty in the posterior samples (figure 8), the spectral combination of (80 + 140 ,w/Sn) achieves the highest AUC among the three, followed by (80 + 140), and then (80 + 120). Another observation is that all three DE strategies incur information loss due to noise in the measurement, as shown by the lower ECS compared to the ICS. Quantitative measures calculated from the decomposed material maps are also consistent with IO findings.

## 6. Discussion

The new IO formulation is based on a DD data acquisition system that maps an imaged object to measurements, both modeled as finite dimensional vectors. Such DD mappings naturally occur in some parametric imaging applications including DE material decomposition. A characteristic of parametric imaging is that the parameters often reside in a low dimensional space; this makes posterior sampling and probabilistic fitting for SOMs considerably simpler. We took advantage of this convenience in this work to demonstrate the working principles of the new, DD-mapping based IO. Despite being low dimensional, our dual energy CT example concerns a realistic nonlinear data acquisition optimization problem, incorporating both signal and background variabilities, for task performance evaluation. The same approach can be applied to many spectral CT systems, such as photon counting with two or more energy bins or multi-energy CT with dual/triple layer detectors.

Very often, the forward models in tomography map object-space image intensity profiles to projection domain line integrals. A DD model for this transformation requires discretization of the image domain using the pixel- or voxel-basis. The CD transformation, that maps the basis function profiles to the data domain, can be absorbed into the DD ‘system matrix.’ The discretization errors in such DD mappings may or may not be problematic for hardware optimization. An example where a DD mapping may be questionable is pinhole size optimization in SPECT (Gross *et al* 2003). The pixel or voxel size then becomes a nuisance parameter that may interfere with the finite pinhole resolution and possibly the IO performance. For other problems, e.g. optimization of energy window for SPECT scatter rejection (Ghaly *et al* 2015), a DD mapping should raise no concerns, as the discretization error in a DD mapping does not play an important role. Then our new IO and the associated large set of computational tools can be applied.

High-dimensional DD mappings for tomographic applications can pose substantial difficulty for both probabilistic characterization of SOMs and image-domain Bayesian posterior sampling. For quadratic problems with MVN image priors, the Bayesian posterior samples can be obtained from multiple image reconstructions, see e.g. (Orieux *et al* 2012, Gilavert *et al* 2014). MVNs as image priors are not adequate to capture the complex patient anatomies. However, significant progress has been made on this issue over the past few years thanks to generative AI. Generative models (GMs) not only can learn the distributions from training data and create samples. Some GMs, e.g., the score-based GMs (Song *et al* 2020), section 4.3, can also compute the *exact* (normalized) likelihood values of data samples via the probability-flow ordinary differential equations. This unique capability makes the density values computable, which can be plugged into our new IO, e.g. (8) and (10), directly. This, together with diffusion posterior sampling techniques (Chung *et al* 2022, Daras *et al* 2024) for Bayesian image reconstruction, makes it possible to characterize IO performance for tasks involving realistic, free-form, patient anatomical variations.

## 7. Summary and conclusions

We propose a new IO formulation for data acquisition task performance optimization that incorporates SOMs. Unlike previous works, we model data acquisition as a DD mapping, which then makes it possible to (i) prescribe PDFs to SOMs, (ii) introduce the notion of ICS of the SOMs due to the stochastic nature of the populations, and (iii) relate the IO performance, i.e., the ECS to ICS via Bayesian posterior sampling.

We validated the new IO formulation using numerical studies including a dual-energy spectral optimization problem. The system performance rank orders obtained by IO agree with physics intuition and quantitative figure of merits.

One advantage of the new IO formulation is that it is conceptually simple, with one expression (8) covering generic binary tasks. Another advantage is that now IO computation can leverage probabilistic inference techniques, many of which, in the context of image formation, are similar to Bayesian image reconstruction. This connection broadens the computational tools available to IO and should make it readily accessible to the image formation community.

Future works may proceed in a few directions. One is to incorporate the latest development in Bayesian inference techniques. Many approximation methods, both deterministic methods Tzikas *et al* (2008) and stochastic sampling based (Blei *et al* 2017), are being actively pursued in both statistics and machine learning community motivated by renewed interest in uncertainty quantification in deep neural networks. Our IO computation can then build on these latest developments and re-purpose them for more efficient and scalable IO computation. Another direction is to apply the new IO to more complex data acquisition optimization problems (Xu and Noo 2024), and to further extend it to binary tasks that involve localization (Khurd and Gindi 2005) or estimation (Clarkson 2007).

## Acknowledgements

This work was supported in part by NIH grants R21 EB033426, R21EB034337, and R21 EB037806. The content is solely the responsibility of the authors and does not necessarily represent the official view of the NIH.

## Data Availability

All data that support the findings of this study are included within the article (and any supplementary information files).
